# Female sex is not associated with worse surgical outcomes in infective endocarditis: a prospective study disproving a common assumption

**DOI:** 10.3389/fcvm.2026.1883895

**Published:** 2026-06-29

**Authors:** N. Pavone, E. M. d’Acierno, G. Mazzenga, F. Cammertoni, M. Calabrese, F. Giovannenze, N. Testa, G. Scoppettuolo, A. Pasquini, M. Grandinetti, E. Romagnoli, G. A. Chiariello, P. Bruno, M. Massetti

**Affiliations:** 1 Department of Cardiovascular Sciences, Fondazione Policlinico Universitario Agostino Gemelli IRCCS, Rome, Italy; 2 Università Cattolica del Sacro Cuore, Rome, Italy; 3 UOC Malattie Infettive-Dipartimento Scienze Mediche e Chirurgiche, Fondazione Policlinico Universitario A. Gemelli IRCCS, Rome, Italy

**Keywords:** cardiac surgery, Endocarditis Team, gender gap, infective endocarditis, sex disparities, surgical outcomes

## Abstract

**Background:**

Sex-based disparities in infective endocarditis (IE) have been increasingly recognized, yet their impact on surgical access and outcomes remains controversial. In particular, whether biological sex influences the likelihood of undergoing surgical treatment and the subsequent prognosis of surgical patients remains a subject of debate.

**Methods:**

This is a single-center, observational, prospective cohort study including 264 consecutive patients diagnosed and treated for IE at a tertiary referral center between January 2023 and December 2025. Among these 264 patients, 194 had an indication for cardiac surgery and 117 finally underwent the operation. Our study included only the group of surgically treated patients. The primary objective was to evaluate the impact of sex on in-hospital mortality following surgery for IE. The secondary objective was to assess the association between sex and mid-term outcomes, defined as a composite endpoint including all-cause mortality, repeat cardiac surgery, hospital readmission for heart failure, or relapse/reinfection of IE within one year from diagnosis. Finally, we investigated whether female sex was associated with reduced surgical access in patients with a formal indication.

**Results:**

A total of 117 patients undergoing cardiac surgery for IE were included: 22 (18.8%) were female. Comorbidities, microbiological etiology and clinical presentation were largely comparable between sexes. The proportion of patients with a formal surgical indication was similar in women and men (67.2% vs. 75.6%, *p* = 0.175). However, women tended to undergo cardiac surgery less frequently than men (48.9% vs. 63.8%). At univariable analysis female sex emerged as a suggestive factor for reduced access to surgery (OR 0.544, *p* = 0.076). Overall in-hospital mortality was 12.8% with no significant sex-related differences (9.1% vs. 13.7% in males, *p* = 0.561). At multivariable analysis, female sex did not emerge as an independent predictor of in-hospital mortality (OR 0.387, *p* = 0.409) nor mid-term outcomes.

**Discussion and Conclusions:**

This study indicates a potential sex-based disparity in surgical access despite similar clinical indications and survival rates. Our findings challenge the assumption that female sex is associated with worse surgical outcomes, highlighting the need for careful individualized clinical judgment and multidisciplinary discussion to prevent potential treatment disparities.

## Graphical Abstract

**Figure:**
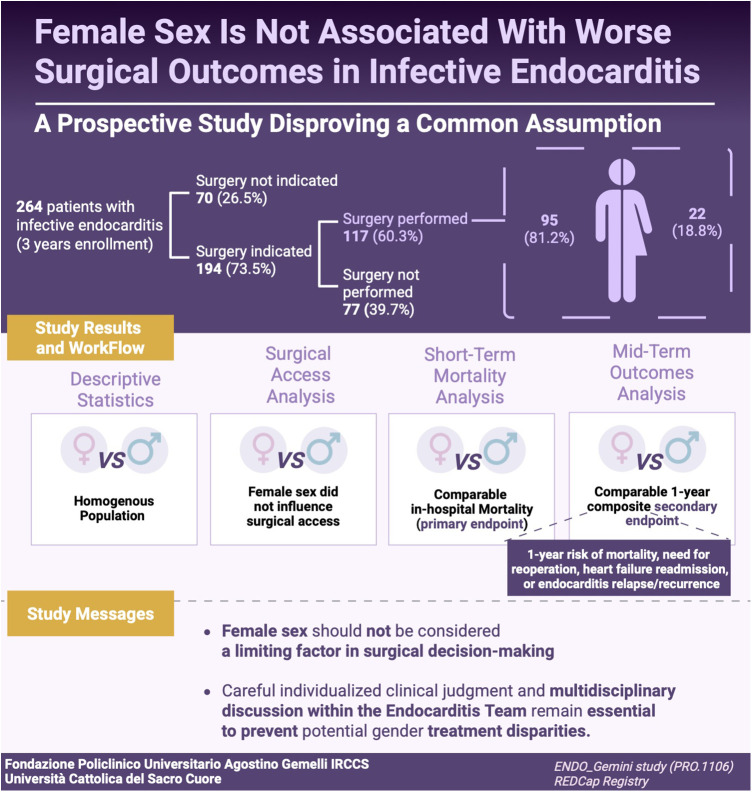

## 1 Introduction

Infective endocarditis (IE) is a life-threatening condition continuing to pose a major challenge to contemporary healthcare systems and imposing a substantial burden of morbidity and mortality (1). Despite advancements in diagnostic techniques and surgical management, clinical outcomes remain suboptimal, with one-year mortality rates exceeding 40% in specific high-risk cohorts (2).

Cardiovascular diseases are established as the primary cause of mortality among women globally (3, 4). Nevertheless, women have been historically underrepresented in major cardiovascular clinical trials, limiting the availability of sex-specific evidence. In recent years, increasing attention has been directed towards the role of sex differences in IE, leading to a growing recognition of significant disparities existing in epidemiology, clinical presentation, management and outcomes (5).

Large population-based studies consistently demonstrate a male predominance in IE incidence across diverse geographic regions and healthcare systems, with age-adjusted incidence rates up to 2.5 times higher than those observed in women (6, 7). Furthermore, women with IE are less likely to undergo valvular surgery despite having comparable evidence-based indications. In the GAMES registry, propensity-score matching for age and surgical risk revealed that women had approximately 25% lower odds of receiving surgical intervention (7). Female patients often present with a higher prevalence of age-associated conditions including anemia, diabetes mellitus and chronic kidney disease potentially contributing to a greater perceived frailty and elevated surgical risk estimates (8).

Existing literature suggests that women may experience distinct patterns of complications and clinical outcomes compared to men. However, data regarding the impact of sex on IE outcomes remain contradictory: several studies have reported higher mortality rates in female patients (9, 10), while others found no significant sex-based differences in early or one-year mortality after risk adjustment (11–13). Interestingly, research into sex differences in operative mortality following cardiac surgery indicates that women often present with worse preoperative functional status and a higher prevalence of depressive syndrome suggesting a potential role for female sex in increasing surgical risk (14, 15).

The aim of our study was to further investigate these controversies by evaluating baseline characteristics, management strategies and clinical outcomes of consecutive patients undergoing cardiac surgery for infective endocarditis at our center over the past three years.

## 2 Methods

### 2.1 Study design and data collection

This is a single-center, observational, prospective cohort study including patients with definite IE diagnosis according to current ESC guidelines (1) treated at our Institution during a 3-years framework. The study was approved by the Local Ethics Committee (ID 7609) and conducted in accordance with the Declaration of Helsinki (1976 and subsequent amendments) and the STROBE guidelines (16, 17). Written informed consent was obtained from all participants. Patient data were collected using REDCap (Research Electronic Data Capture), a secure web-based software platform designed for building and managing online surveys and databases. The REDCap system allows standardized data entry through customizable electronic case report forms (eCRFs), ensures data quality through validation rules and audit trails, and supports secure data storage compliant with institutional and regulatory requirements.

The dedicated REDCap registry was specifically implemented to support an institutional clinical care pathway for patients with IE (PRO.1106, ENDO_Gemini study), established in 2023 in our Hospital (18). This clinical pathway was designed to optimize the management of patients with IE across all stages of care, from early diagnosis to standardized antimicrobial and surgical treatment strategies according to the framework of the Endocarditis Team model recommended by the ESC guidelines.

All patients managed by our institutional Endocarditis Team were consecutively enrolled in the REDCap registry. Data on patients' baseline clinical characteristics and presentation of IE were systematically collected. Major risk stratification scores for IE were recorded in the study database including EuroSCORE II, IE-STS, APORTEI, De Feo, and Risk-E scores. Microbiological, echocardiographic, and radiological findings were integrated to assess disease severity. Treatment strategies were also systematically recorded. When cardiac surgery was indicated, detailed data on surgical procedures were described. In patients with a guideline-based indication for surgery who did not ultimately undergo surgical intervention, the reasons for non-performance were documented. Follow-up data included scheduled visits with infectious disease specialists, carried out up to 1 year after surgical or conservative management.

In this study, we focused on patients who underwent cardiac surgery as decided by the local Endocarditis Team. The primary endpoint was to evaluate the impact of sex on in-hospital mortality following surgery for IE. The secondary objective was to assess the association between sex and mid-term outcomes, defined as a composite endpoint including all-cause mortality, repeat cardiac surgery, hospital readmission for heart failure, or relapse/reinfection of IE within one year from the primary diagnosis. Finally, we investigated whether female sex was associated with reduced surgical access in patients with a formal indication, adjusting for potential predictors of inoperability such as age, CCI, dementia, BMI < 18, LVEF < 30%, TAPSE < 14 mm, neurologic risk, previous cardiac or transcatheter interventions, intravenous drug use, and *Staphylococcus aureus* infections.

### 2.2 Statistical analysis

Descriptive statistics summarized clinical and demographic data. Qualitative variables were expressed as frequencies, while quantitative variables were presented as medians ± IQR, as the Shapiro–Wilk test assessed no Gaussian distribution. Differences between genders were tested with the Fisher-Freeman-Halton exact test or Chi-squared test for qualitative variables, as appropriate, and Mann–Whitney *U*-test for quantitative variables. Potential predictors of mortality were assessed with logistic regression models, reporting ORs and 95% CIs. Statistical significance was set at *p* < 0.05. Variables showing statistically significant or suggestive (0.05 ≤ *p* < 0.10) associations in the univariable analysis were included in the multivariable model to identify potential independent predictors of mortality, in line with van Smeden and TRIPOD recommendations (17, 19). In addition, survival analysis was performed to evaluate time-to-event outcomes. Survival probabilities over time were estimated using the Kaplan–Meier method, and differences between groups were assessed using the log-rank test.

The analysis was performed on only surgically operated patients for in-hospital mortality (primary end-point), and for the composite secondary end-point. The Hosmer-Lemeshow test assessed goodness-of-fit. The c-index (AUC) quantified model accuracy, and corrected Dxy measured model optimism. Internal validation used bootstrap resampling (1,000 repetitions) and calibration plots, illustrating predicted vs. observed outcomes. Surgical access logistic regression analysis was carried out with analogous methodology on all patients with formal indication to surgery.

All statistical analysis was performed in Python (version 3.12.13), using Pandas and NumPy for data management and preprocessing, and Statsmodels, Pingouin, and Lifelines for statistical modeling and survival analysis.

## 3 Results

**Figure 1:**
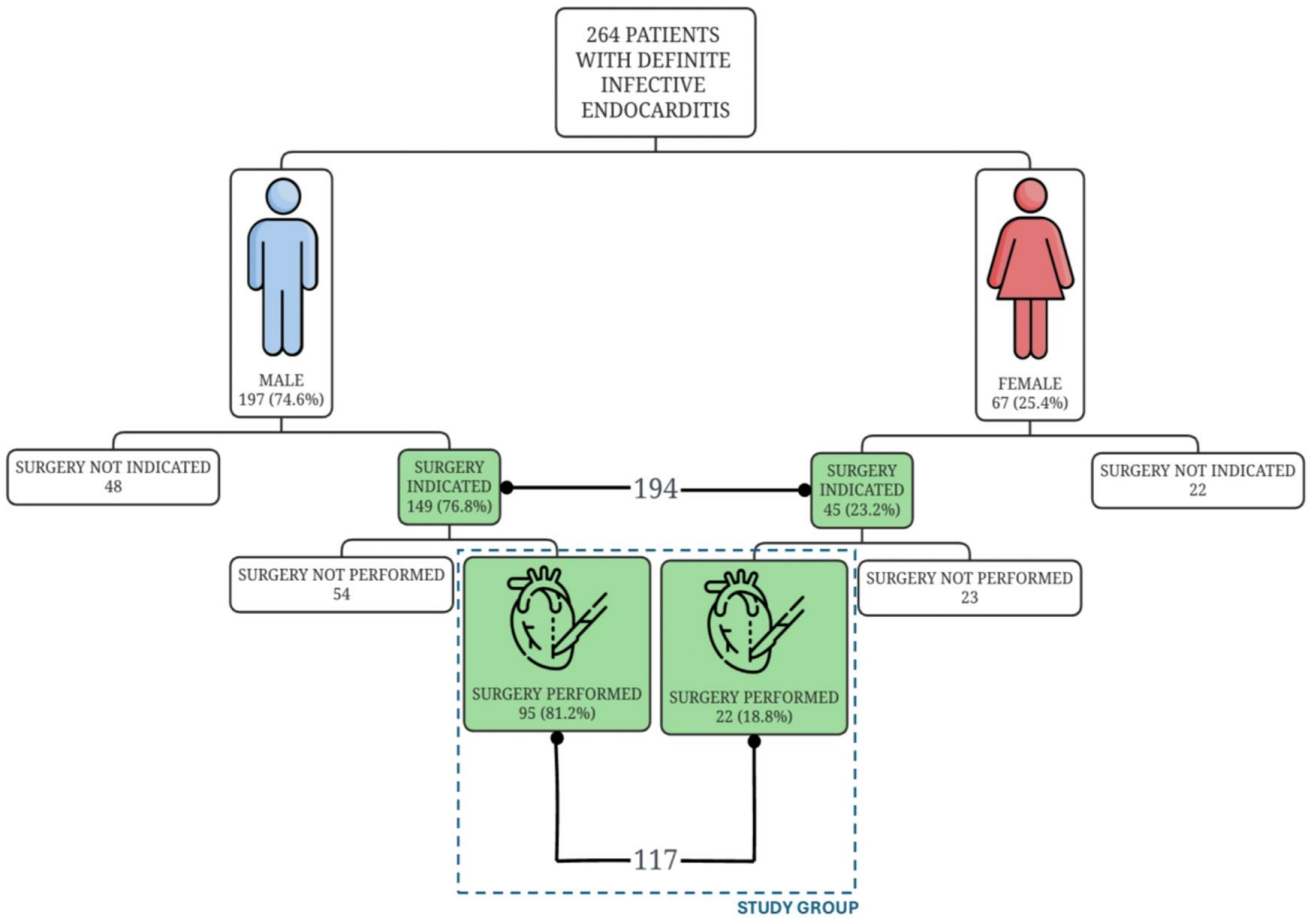
Treatment Algorithm of the study population.

Between January 2023 and December 2025, a total of 264 patients with diagnosis of definite IE, according to current ESC guideline criteria (1), were consecutively enrolled. Female patients accounted for 67 cases (25.4%), whereas 197 patients (74.6%) were males. Among the 264 patients with IE, 194 patients had a guideline-based indication for surgery [45 women (23.2%) and 149 men (76.8%)]. Of these, 117 patients effectively underwent cardiac surgery [22 women (48.89%) vs. 95 men (63.76%)]. The treatment algorithm is summarized in Figure 1.

To ensure clarity, we first present the findings regarding access to surgery among all indicated patients, before focusing specifically on the surgically managed study cohort.

### 3.1 Patients with surgical indication

Overall, surgical indications (heart failure, uncontrolled infection and prevention of embolism) and timing (elective, urgent, or emergent) were comparable between sexes. However, while indications for surgery related to embolic risk prevention were similarly distributed between female and male patients (68.4% vs. 65.2%, *p* = 0.794), different patterns emerged for other surgical indications: heart failure tended to be more frequent among women (31.6% vs. 14.7%, *p* = 0.093), whereas uncontrolled infection was more commonly observed among male patients (44.1% vs. 26.3%, *p* = 0.162). Results are shown in Table 1.

**Table 1:** Surgical indications of our surgical cohort (*n* = 117).

Variables	Overall (*N* = 117)	Female (*N* = 22)	Male (*N* = 95)	*p* Value
Timing Elective, *n* (%)	44 (37.6)	9 (40.9)	35 (36.9)	0.734
Timing Urgent, *n* (%)	70 (59.8)	12 (54.5)	58 (61.1)	
Timing Emergent, *n* (%)	3 (2.6)	1 (4.5)	2 (2.1)	
Diagnosis-to-surgery (days), Mdn (IQR)	12 (6–26)	17 (4–29.3)	11 (6.5–23.5)	0.608
Appropriate Surgical Timing, *n* (%)	96 (82.1)	16 (72.7)	80 (84.2)	0.206
Non-elective Indication Reason: Heart Failure, *n* (%)	16 (18.4)	6 (31.6)	10 (14.7)	0.093
Non-elective Indication Reason: Uncontrolled Infection, *n* (%)	35 (40.2)	5 (26.3)	30 (44.1)	0.162
Non-elective Indication Reason: Embolism, *n* (%)	58 (65.9)	13 (68.4)	45 (65.2)	0.794

Despite surgical indication, female patients tended to undergo surgery less frequently (48.9% vs. 63.8%, *p* = 0.074). In univariable analysis female sex emerged as a suggestive factor for reduced access to surgery (OR 0.544, *p* = 0.076). However, in the multivariable model, female sex was no longer an independent predictor. Instead, higher CCI [OR 0.751 (0.629–0.898), *p* = 0.002], dementia [OR 0.195 (0.038–0.999), *p* = 0.050], neurologic risk [OR 0.460 (0.229–0.922), *p* = 0.029], and *Staphylococcus aureus* infections [OR 0.312 (0.136–0.7149), *p* = 0.006] were independent factors associated with lower likelihood of surgical intervention. Results are shown in Table 2.

**Table 2:** Predictors of surgical access among all patients with formal surgical indication (*n* = 194).

Variables	Surgery performed (*n* = 117)	Surgery unperformed (*n* = 77)	Logistic regression analysis OR (95%CI); *p*	Multivariable analysis OR (95%CI); *p*
Female Gender, *n* (%)	22 (18.8)	23 (29.9)	0.544 (0.277–1.066); 0.076	0.721 (0.005–98.319); 0.896
Age, Mdn(IQR)	66 (55–71)	76 (64.5–81.5)	0.945 (0.919–0.970); <0.001	0.979 (0.941–1.018); 0.289
Female Gender × Age				0.995 (0.927–1.067); 0.881
CCI, Mdn(IQR)	4 (2–5)	5 (4–8)	0.681 (0.588–0.787); <0.001	**0.751 (0.629–0.898); 0.002**
Dementia, *n* (%)	2 (1.7)	12 (15.6)	0.094 (0.020–0.434); 0.020	**0.195 (0.038–0.999); 0.050**
BMI <18, *n* (%)	3 (2.6)	4 (5.2)	0.491 (0.107–2.258); 0.361	
Intravenous Drugs use, *n* (%)	11 (9.4)	2 (2.6)	3.892 (0.838–18.069); 0.083	2.030 (0.357–11.537); 0.425
IE-STS score M&M, Mdn(IQR)	22 (15–29)	23 (16–34)	0.981 (0.925–1.041); 0.526	
Aportei Risk Score, Mdn(IQR)	45 (30–62)	49.5 (39–67.5)	0.241 (0.984–0.957); 0.241	
FE < 30%, *n* (%)	4 (3.4)	6 (7.8)	0.419 (0.114–1.536); 0.189	
TAPSE < 14 mm, *n* (%)	17 (14.5)	12 (15.6)	0.921 (0.413–2.054); 0.840	
previous cardiac surgery, *n* (%)	41 (35)	31 (40.3)	0.801 (0.442–1.448); 0.462	
previous TAVI, *n* (%)	6 (5.1)	6 (7.8)	0.640 (0.198–2.061); 0.454	
CerebroVascular Accidents and SNC Emboli, *n* (%)	39 (33.3)	40 (51.9)	0.463 (0.256–0.834); 0.010	**0.460 (0.229–0.922); 0.029**
*S.aureus* Infective Endocarditis, *n* (%)	18 (15.4)	25 (32.5)	0.378 (0.189–0.756); 0.006	**0.312 (0.136–0.714); 0.006**
Previous endocarditis, *n* (%)			2.056 (0.538–7.849); 0.292	

Bold values indicate statistically significant differences (*p* < 0.05).

### 3.2 Surgically managed patients

#### 3.2.1 IE presentation and preoperative risk assessment

Baseline characteristics and clinical presentation are summarized in Table 3. The median age was 66 years (IQR 55–71) with no significant differences between sexes [67 years (IQR 60–73.25) vs. 64 years (IQR 55–71) respectively; *p* = 0.241]. No significant sex-related differences were observed in most comorbidities, either individually or according to the Charlson Comorbidity Index (CCI). Female patients were characterized by significantly lower body surface area [1.8 m^2^ (1.6–1.925) vs. 1.9 m^2^ (1.7–2) in males, *p* = 0.011], showed a higher prevalence of prior transcatheter valve implantation (13.6% vs. 3.2%, *p* = 0.048) and more frequently presented with mitral valve involvement (54.5% vs. 29.5%, *p* = 0.025). No significant sex-related differences were observed in the prevalence of prosthetic valve endocarditis (36.4% vs. 34.7%, *p* = 0.885) or in the distribution of causative pathogens, as shown in Table 4. Similarly, the incidence of IE-related complications did not differ significantly between groups.

**Table 3:** General characteristics and clinical features of our surgical cohort (*n* = 117).

Variables	Overall (*N* = 117)	Female (*N* = 22)	Male (*N* = 95)	*p* Value
Age (years), Mdn (IQR)	66 (55–71)	67 (60–73.25)	64 (55–71)	0.241
Weight (kg), Mdn (IQR)	73 (65–84.5)	70 (58.25–80)	74 (65–85)	0.102
Height (cm), Mdn (IQR)	170 (165–177.5)	160 (155–165.75)	172 (170–179)	**<0.001**
BMI (kg/m^2^), Mdn (IQR)	25 (22.8–27.45)	25.6 (22.6–31.3)	24.6 (22.8–27.2)	0.225
BSA	1.9 (1.7–2)	1.8 (1.6–1.93)	1.9 (1.7–2)	**0.011**
Congestive Heart Failure, *n* (%)	23 (19.8)	5 (22.7)	18 (19.1)	0.705
CerebroVascular Accidents, *n* (%)	11 (9.5)	1 (4.8)	10 (10.5)	0.415
Dementia, *n* (%)	2 (1.7)	0 (0)	2 (2.1)	1.000
COPD, *n* (%)	14 (12)	4 (18.2)	10 (10.5)	0.319
Myocardial infarction, *n* (%)	14 (12.3)	1 (4.5)	13 (14.1)	0.219
Diabetes Mellitus, *n* (%)	27 (23.1)	6 (27.3)	21 (22.1)	0.267
Atrial Fibrillation, *n* (%)	26 (22.2)	6 (27.3)	20 (21.1)	0.527
Intravenous Drug Use, *n* (%)	11 (9.6)	1 (4.5)	10 (10.8)	0.373
CKD, *n* (%)	11 (9.5)	4 (18.2)	7 (7.4)	0.122
Hemodialysis, *n* (%)	3 (2.6)	2 (9.5)	1 (1.1)	0.087
CCI, Mdn(IQR)	4 (3–6)	5 (3–6)	4 (2–6)	0.372
Previous Heart Valve Surgery, *n* (%)	41 (35.7)	7 (31.8)	34 (36.6)	0.676
Previous Transcatheter Valve Implantation, *n* (%)	6 (5.2)	3 (13.6)	3 (3.2)	0.048
Previous Endocarditis, *n* (%)	9 (7.8)	1 (4.5)	8 (8.6)	0.524
LVEF (%), Mdn(IQR)	55 (50–61)	57 (52–69)	55 (50–61)	0.179
TAPSE (mm), Mdn(IQR)	20 (16–24)	20 (13–25.75)	20 (16–24)	0.598
Aortic Stenosis >=moderate, *n* (%)	15 (12.8)	4 (18.2)	11 (11.6)	0.404
Aortic Regurgitation >=moderate, *n* (%)	50 (42.7)	6 (27.3)	44 (46.3)	0.104
Mitral Stenosis >=moderate, *n* (%)	8 (6.8)	2 (9.1)	6 (6.3)	0.642
Mitral Regurgitation >=moderate, *n* (%)	42 (35.9)	12 (54.5)	30 (31.6)	**0.043**

Bold values indicate statistically significant differences (*p* < 0.05).

**Table 4:** Endocarditis features and complications of our surgical cohort (*n* = 117).

Variables	Overall (*N* = 117)	Female (*N* = 22)	Male (*N* = 95)	*p* Value
NVE, *n* (%)	75 (64.1)	16 (72.7)	59 (62.1)	0.349
PVE, *n* (%)	41 (35)	8 (36.4)	33 (34.7)	0.885
CIED-IE, *n* (%)	6 (5.1)	2 (9.1)	4 (4.2)	0.350
Aortic, *n* (%)	39 (33.3)	6 (27.3)	33 (34.7)	0.503
Mitral, *n* (%)	40 (34.2)	12 (54.5)	28 (29.5)	**0.025**
Tricuspid, *n* (%)	4 (3.4)	4 (4.2)	0 (0)	1.000
Pathogen—MSSA, *n* (%)	17 (17.2)	3 (18.8)	14 (16.9)	0.847
Pathogen—MRSA, *n* (%)	1 (1)	1 (6.3)	0 (0)	
Pathogen—Coag neg Staphylococci, *n* (%)	1 (1)	0 (0)	1 (1.2)	
Pathogen—Streptococcal spp, *n* (%)	25 (25.2)	6 (37.5)	19 (22.9)	
Pathogen—Enterococcal spp, *n* (%)	21 (21.2)	1 (6.3)	20 (24.1)	
Acute Heart Failure, *n* (%)	17 (14.8)	4 (18.2)	13 (14)	0.617
Cardiogenic Shock, *n* (%)	3 (2.6)	1 (4.5)	2 (2.2)	0.474
Sepsis, *n* (%)	19 (16.5)	4 (18.2)	15 (16.1)	0.816
Septic Shock, *n* (%)	3 (2.6)	1 (9.5)	1 (1.1)	0.087
CNS Emboli, *n* (%)	31 (27.2)	4 (18.2)	27 (29.3)	0.290
Splenic Emboli, *n* (%)	42 (36.5)	6 (27.3)	36 (38.7)	0.316
Hepatic Emboli, *n* (%)	11 (9.6)	3 (13.6)	8 (8.6)	0.470
Lung Emboli, *n* (%)	6 (5.2)	1 (4.5)	5 (5.4)	1.000
Kidney Emboli, *n* (%)	25 (21.7)	5 (22.7)	20 (21.5)	0.901
Gut Emboli, *n* (%)	1 (0.9)	0 (0)	1 (1.1)	1.000
Abscess, *n* (%)	24 (21.8)	4 (20)	20 (22.2)	0.828
Fistula, *n* (%)	9 (8.1)	1 (5)	8 (8.8)	0.574
Valve Perforation, *n* (%)	21 (18.9)	5 (25)	16 (17.6)	0.443
Pseudoaneurysm, *n* (%)	15 (13.6)	3 (15)	12 (13.3)	0.844

Bold values indicate statistically significant differences (*p* < 0.05).

IE-STS morbidity and mortality score was significantly higher in females compared with males (29.5 vs. 21%, *p* = 0.004). Even though not reaching statistical significance, women tended to have higher median Aportei scores [median 53 (42–68.5) vs. 44 (26–60.5), *p* = 0.090]. Other scoring systems showed overall comparable risk profiles between sexes. Complete risk stratification among the two groups is shown in Table 5.

**Table 5:** Risk assessment of our surgical cohort (*n* = 117).

Variables	Overall (*N* = 117)	Female (*N* = 22)	Male (*N* = 95)	*p* Value
De Feo risk score, Mdn (IQR)	12 (9–16)	13.5 (10.5–16.5)	11.5 (9–15.75)	0.432
De Feo class 3 or 4, *n* (%)	18 (15.4)	3 (13.6)	15 (15.8)	0.801
Aportei risk score, Mdn (IQR)	45 (30–62)	53 (42–68.5)	44 (26–60.5)	0.090
Aportei class high or extreme, *n* (%)	32 (27.4)	8 (36.4)	24 (25.3)	0.293
Risk E, Mdn (IQR)	18.5 (13–24)	20 (13.5–25)	16 (13–24)	0.320
IE mortality, Mdn (IQR)	10 (7–12)	10 (6.5–12)	10 (7–12)	0.892
IE 6 m mortality, Mdn (IQR)	30.2 (17.4–39.8)	30.2 (15.5–39.9)	30.2 (17.4–39.8)	0.968
IE STS mortality, Mdn (IQR)	28 (21–36)	33.5 (22.5–35.75)	27.5 (21–36)	0.304
IE STS M&M, Mdn (IQR)	22 (15–29)	29.5 (22–34)	21 (13–25)	**0.004**

Bold values indicate statistically significant differences (*p* < 0.05).

#### 3.2.2 Outcomes

Women more frequently underwent mitral and tricuspid valve repair compared with men (MVr 27.3% vs. 3.2%, *p* < 0.001; TVr 36.4% vs. 16.8%, *p* = 0.041). Postoperative conduction disturbances were also more common among females, with higher rates of third-degree AV block (22.7% vs. 7.4%, *p* = 0.048) and a trend toward more pacemaker implantations (13.6% vs. 3.2%, *p* = 0.079). Other surgical and postoperative outcomes including operative times, intensive care unit (ICU) stay, reintubation, acute kidney injury and need for dialysis, early bleeding requiring surgical revision, and ICU mortality were comparable between groups, as shown in Table 6.

**Table 6:** Surgical and early post-operative data (*n* = 117).

Variables	Overall (*N* = 117)	Female (*N* = 22)	Male (*N* = 95)	*p* Value
AVR, *n* (%)	65 (55.6)	9 (40.9)	56 (58.9)	0.125
MVR, *n* (%)	46 (39.3)	10 (45.5)	36 (37.9)	0.513
TVR, *n* (%)	7 (6)	0 (0)	7 (7.4)	0.189
Bentall, *n* (%)	6 (5.1)	1 (4.5)	5 (5.3)	0.891
MVPl, *n* (%)	9 (7.7)	6 (27.3)	3 (3.2)	<**0.001**
TVPl, *n* (%)	24 (20.5)	8 (36.4)	16 (16.8)	**0.041**
CABG, *n* (%)	9 (7.7)	1 (4.5)	8 (8.4)	0.539
PMK implantation intraop, *n* (%)	7 (6)	0 (0)	7 (7.4)	0.189
Pericardial Patch, *n* (%)	17 (14.5)	2 (9.1)	15 (15.8)	0.422
Lead Extraction, *n* (%)	4 (3.4)	0 (0)	4 (4.2)	1.000
ACC time, Mdn(IQR)	107 (80–126)	89 (80–113)	109.5 (80–135)	0.151
CEC time, Mdn (IQR)	130 (106–169)	114 (103–143)	135 (107–170)	0.227
Surgery time, Mdn (IQR)	307 (239–409)	268 (216–318)	320 (256–410)	0.064
Secondary ACC, *n* (%)	4 (3.5)	1 (4.8)	3 (3.2)	0.562
Secondary CEC, *n* (%)	5 (4.4)	2 (9.5)	3 (3.3)	0.232
ECMO, *n* (%)	3 (2.6)	0 (0)	3 (3.2)	1.000
ICU LoS (days), Mdn (IQR)	3 (2–6)	2 (2–5.5)	3 (2–6)	0.189
Post-op Bleeding, *n* (%)	25 (21.4)	3 (13.6)	22 (23.2)	0.326
Surgical Revision, *n* (%)	11 (9.4)	1 (4.5)	10 (10.5)	0.386
Gauze Closure, *n* (%)	17 (14.5)	3 (13.6)	14 (14.7)	0.895
Intubation hours, Mdn (IQR)	16 (10.8–30.5)	16 (9–48)	16 (11–23)	0.566
Reintubation, *n* (%)	11 (9.4)	1 (4.5)	10 (10.5)	0.386
Tracheostomy, *n* (%)	2 (1.7)	1 (4.5)	1 (1.1)	0.255
Ventricular Fibrillation, *n* (%)	1 (0.9)	0 (0)	1 (1.1)	1.000
Tamponade, *n* (%)	4 (3.4)	1 (4.5)	3 (3.2)	0.572
Cardiac Arrest, *n* (%)	5 (4.3)	1 (4.5)	4 (4.2)	1.000
III degree AV block, *n* (%)	12 (10.3)	5 (22.7)	7 (7.4)	**0** **.** **048**
PMK implantation, *n* (%)	6 (5.1)	3 (13.6)	3 (3.2)	0.079
CVVHDF, *n* (%)	7 (6)	1 (4.5)	6 (6.3)	0.752
IRRT, *n* (%)	4 (3.4)	2 (9.1)	2 (2.1)	0.106
Gastrointestinal Complication, *n* (%)	2 (1.7)	1 (4.5)	1 (1.1)	0.342
ICU readmission, *n* (%)	2 (1.7)	1 (4.5)	1 (1.1)	0.342
Intra-operative mortality, *n* (%)	2 (1.7)	0 (0)	2 (2.1)	1.000
ICU-mortality, *n* (%)	11 (9.4)	2 (9.1)	9 (9.5)	0.956

Bold values indicate statistically significant differences (*p* < 0.05).

**Figure 2:**
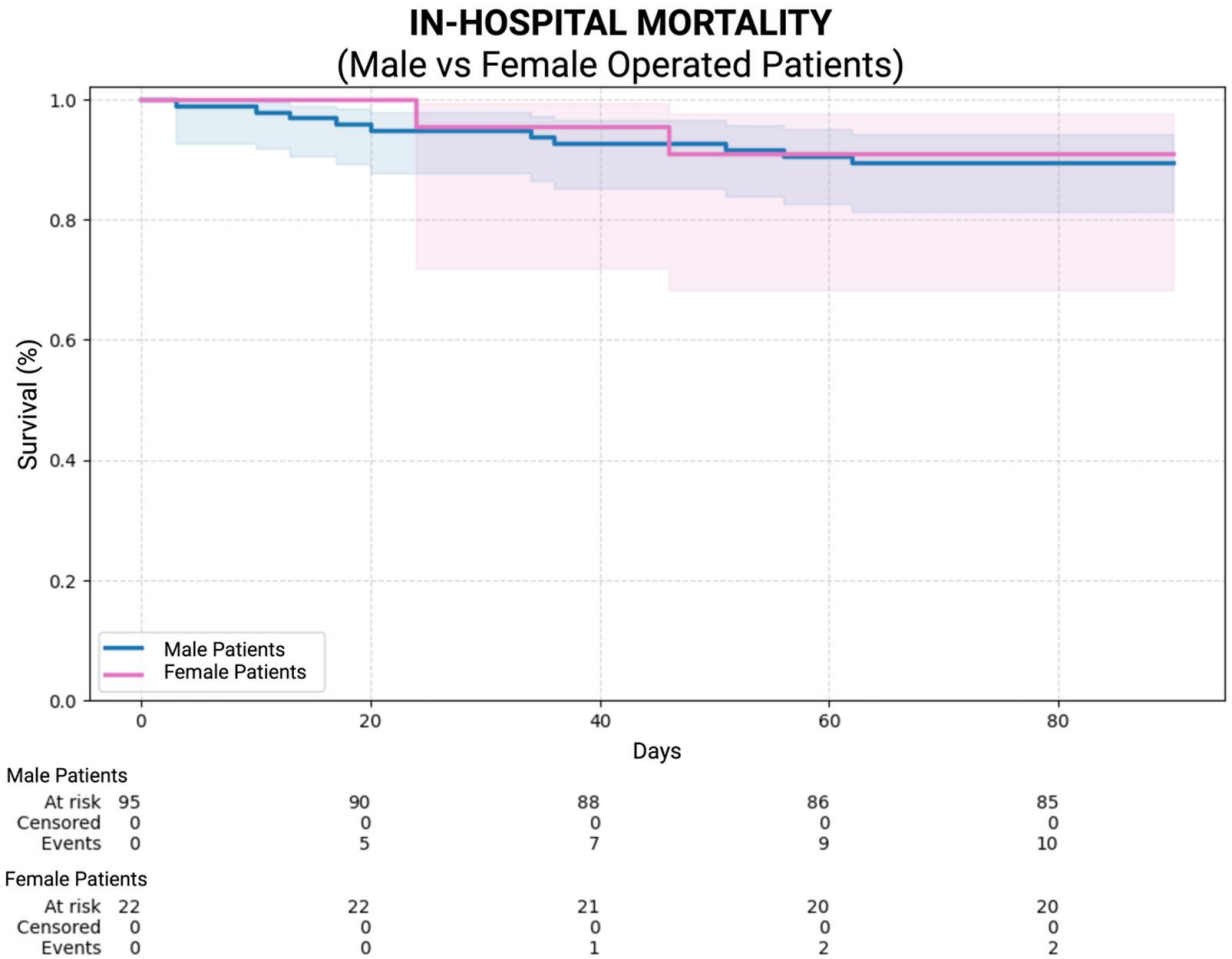
Kaplan–meier survival curves for in-hospital mortality stratified by sex.

Overall in-hospital mortality was 12.8% with no significant sex-related differences (9.1% vs. 13.7% in males, *p* = 0.561), as further delineated by the Kaplan–Meier survival analysis (Figure 2). Similar trends were observed for 90 days (9.1% vs. 13.7% in males, *p* = 0.561), 6 months (9.1% vs. 15.8% in males, *p* = 0.421) and 1-year mortality (9.1% vs. 16.8% in males, *p* = 0.364). The corresponding Kaplan–Meier survival curves are presented in Supplementary Figures S1, S2. No differences were found regarding in-hospital length of stay (median stay, days 46.5 vs. 38.5 in males, *p* = 0.221), hospital readmission (22.7% vs. 14.7% in males, *p* = 0.360), and 1-year IE relapse or reinfection. Results are shown in Table 7.

**Table 7:** Outcomes of our surgical cohort (*n* = 117).

Variables	Overall (*N* = 117)	Female (*N* = 22)	Male (*N* = 95)	*p* Value
LoS (days), Mdn (IQR)	40 (31–56)	46.5 (32.7–65.5)	38.5 (30.25–55)	0.221
In-hospital mortality, *n* (%)	15 (12.8)	2 (9.1)	13 (13.7)	0.561
90d Mortality, *n* (%)	15 (12.8)	2 (9.1)	13 (13.7)	0.561
6 m Mortality, *n* (%)	17 (14.5)	2 (9.1)	15 (15.8)	0.421
1y Mortality, *n* (%)	18 (15.4)	2 (9.1)	16 (16.8)	0.364
Age-stratified 1y Mortality: <61 years, *n* (% 1y-dead patients)	5 (27.8)	1 (50)	4 (25)	0.317
Age-stratified 1y Mortality: 61–78 years, *n* (%1y-dead patients)	11 (61.1)	1 (50)	10 (62.5)	0.739
Age-stratified 1y Mortality: pt > 78 years, *n* (%1y-dead patients)	2 (1.1)	0 (0)	2 (12.5)	1.000
Cause of death: cardiovascular, *n* (%)	12 (10.3)	2 (9.1)	10 (10.5)	0.842
Cause of death: non-cardiovascular, *n* (%)	4 (3.4)	0 (0)	4 (4.2)	0.327
Hospital Readmission, *n* (%)	19 (16.2)	5 (22.7)	14 (14.7)	0.360
Hosp Readm for HF, *n* (%)	1 (0.9)	1 (4.5)	0 (0)	0.188
New Cardiac Surgery, *n* (%)	4 (3.4)	0 (0)	4 (4.2)	1.000
1y IE Relapse, *n* (%)	3 (2.6)	0 (0)	3 (3.2)	1.000
1y IE Reinfection, *n* (%)	6 (5.1)	0 (0)	6 (6.3)	0.592
Persistent Bacteremia, *n* (%)	2 (1.7)	0 (0)	2 (2.1)	1.000
Bacteremia Relapse, *n* (%)	1 (0.9)	0 (0)	1 (1.1)	1.000
New embolic events, *n* (%)	3 (2.6)	0 (0)	3 (3.2)	1.000

To evaluate whether female sex represented an actual risk factor for in-hospital mortality, we performed logistic regression analyses including sex and other clinically relevant predictors of poor outcomes. At univariable and multivariable analysis, female sex did not emerge as an independent predictor of in-hospital mortality [OR 0.631 (0.132–3.022), *p* = 0.564; OR 0.387 (0.041–3.684), *p* = 0.409]. Factors independently associated with in-hospital mortality were preoperative sepsis [OR 16.018 (1.261–203.41); *p* = 0.032] and diagnosis-to-surgery time [OR 0.877 (0.777–0.990); *p* = 0.033]. Included variables and results are shown in Table 8. Pearson correlation matrix for potential predictors is displayed in Supplementary Figure S3.

**Table 8:** Predictors of in-hospital mortality among our surgical cohort (*n* = 117).

Variables	In-hospital mortality (*n* = 15)	In-hospital survival (*n* = 102)	Logistic Regression Analysis OR (95%CI); *p*	Multivariable Analysis OR (95%CI); p
Female Gender, *n* (%)	2 (13.3)	20 (19.6)	0.631 (0.132–3.022); 0.564	0.387 (0.041–3.684); 0.409
Age, Mdn(IQR)	67 (55.50–70.25)	66 (58–72)	1.014 (0.972–1.058); 0.526	
CCI, Mdn(IQR)	5 (3.25–6.75)	4 (2–5)	1.169 (0.942–1.449); 0.156	
IE-STS score M&M, Mdn(IQR)	23 (21–29)	17.50 (11–28.50)	1.027 (0.940–1.122); 0.551	
Aportei risk score, Mdn(IQR)	56 (38–69)	33 (25.25–55.75)	1.013 (0.977–1.050); 0.494	
FE < 50%, *n* (%)	4 (26.7)	23 (22.5)	1.249 (0.363–4.295); 0.724	
FE < 30%, *n* (%)	1 (6.7)	3 (2.9)	2.357 (0.229–24.259); 0.471	
TAPSE < 17 mm, *n* (%)	6 (40)	25 (24.5)	2.053 (0.665–6.339); 0.211	
TAPSE < 14 mm, *n* (%)	3 (20)	14 (13.7)	1.571 (0.393–6.279); 0.522	
Acute Heart Failure, *n* (%)	3 (20)	14 (13.7)	1.571 (0.393–6.279); 0.522	
Cardiogenic Shock, *n* (%)	1 (6.7)	2 (2)	3.571 (0.304–42.002); 0.311	
Sepsis, *n* (%)	7 (46.7)	12 (11.8)	**6.562 (2.017–21.351); 0.002**	**16.018 (1.261–203.410); 0.032**
Septic Shock, *n* (%)	1 (6.7)	2 (2)	3.571 (0.304–42.002); 0.311	
CerebroVascular Accidents and CNS Emboli, *n* (%)	5 (33.3)	34 (33.3)	1.000 (0.317–3.157); 1.000	
S.Aureus Infective Endocarditis	4 (26.7)	4 (13.7)	2.286 (0.638–8.186); 0.204	
previous cardiac surgery, *n* (%)	8 (53.3)	33 (32.4)	2.390 (0.799–7.150); 0.119	
ET discussion performed, *n* (%)	9 (60)	62 (60.8)	0.968 (0.320–2.927); 0.954	
Diagnosis-to-surgery (days), Mdn (IQR)	11 (7.75–15)	14 (4–29)	0.920 (0.834–1.014); 0.092	**0.877 (0.777–0.990); 0.033**
Appropriate Surgical Timing, *n* (%)	12 (80)	84 (82.4)	0.857 (0.219–3.352); 0.825	
Local complications (abcesses, fistulae or pseudoaneurysms), *n* (%)	6 (40)	27 (26.5)	1.333 (0.670–2.649); 0.413	

Bold values indicate statistically significant differences (*p* < 0.05).

Furthermore, univariable analysis showed no significant association between female sex and the composite secondary endpoint [OR 0.442 (0.120–1.623); *p* = 0.219]. In contrast, preoperative sepsis [OR 2.836 (1.008–7.983); *p* = 0.048] and the CCI [OR 1.228 (1.016–1.483); *p* = 0.033] appeared to have a greater impact, although they subsequently lost statistical significance in the multivariable model. All results are shown in Table 9.

**Table 9:** Predictors of composite secondary end-point (1y mortality + New Cardiac Surgery + Hosp Readm for HF + IE Relapse or Reinfection) among our surgical cohort (*n* = 117).

Variables	Secondary endpoint YES (*n* = 28)	Secondary endpoint NO (*n* = 89)	Logistic Regression Analysis OR (95%CI); *p*	Multivariable Analysis OR (95%CI); *p*
Female Gender, *n* (%)	3 (10.7)	19 (21.3)	0.442 (0.120–1.623); 0.219	0.227 (0.035–1.477; 0.121
Age, Mdn(IQR)	67 (56.50–70.25)	66 (55–72.50)	1.024 (0.986–1.063); 0.215	
CCI, Mdn(IQR)	5.50 (3.75–7)	4 (2–4.50)	**1.228 (1.016–1.483); 0.033**	1.309 (0.938–1.828); 0.114
IE-STS score M&M, Mdn(IQR)	24 (21–30)	18 (11–25.50)	1.009 (0.941–1.082); 0.800	
Aportei risk score, Mdn(IQR)	56 (40.50–69.50)	33 (22–63.25)	1.001 (0.974–1.029); 0.935	
FE <50%, *n* (%)	8 (28.6)	19 (21.3)	1.474 (0.562–3.864); 0.430	
FE <30%, *n* (%)	1 (3.6)	3 (3.4)	1.062 (0.106–10.633); 0.959	
TAPSE < 17 mm, *n* (%)	9 (32.1)	22 (24.7)	1.443 (0.570–3.648); 0.439	
TAPSE < 14 mm, *n* (%)	6 (21.4)	11 (12.4)	1.934 (0.643–5.818); 0.241	
Acute Heart Failure, *n* (%)	5 (17.9)	12 (13.5)	1.395 (0.445–4.373); 0.568	
Cardiogenic Shock, *n* (%)	2 (7.1)	1 (1.1)	6.769 (0.590–77.663); 0.125	
Sepsis, *n* (%)	8 (28.6)	11 (12.4)	**2.836 (1.008–7.983); 0.048**	3.005 (0.437–20.651); 0.263
Septic Shock, *n* (%)	1 (3.6)	2 (2.2)	1.611 (0.141–18.465); 0.702	
CerebroVascular Accidents and CNS Emboli, *n* (%)	7 (25)	32 (36)	0.594 (0.228–1.549); 0.287	
S.Aureus Infective Endocarditis, *n* (%)	5 (17.9)	13 (14.6)	1.271 (0.410–3.942); 0.678	
previous cardiac surgery, *n* (%)	12 (42.9)	29 (32.6)	1.552 (0.650–3.703); 0.322	
ET discussion performed, *n* (%)	16 (57.1)	55 (61.8)	0.824 (0.348–1.952); 0.660	
Diagnosis-to-surgery (days), Mdn (IQR)	11.50 (9.25–14.50)	14 (4–32)	0.953 (0.903–1.006); 0.080	0.946 (0.888–1.007); 0.082
Appropriate Surgical Timing, *n* (%)	22 (78.6)	74 (83.1)	0.743 (0.258–2.145); 0.583	
Local complications (abcesses, fistulae or pseudoaneurysms), *n* (%)	8 (28.6)	25 (28.1)	1.096 (0.609–1.971); 0.760	

Bold values indicate statistically significant differences (*p* < 0.05).

## 4 Discussion

Despite substantial advances in diagnostic modalities, surgical techniques, and perioperative management, IE remains a life-threatening condition, challenging healthcare systems and imposing a considerable burden of morbidity and mortality, particularly among high-risk patients. Even though cardiovascular disease in women has recently received increasing scientific attention, the knowledge gap regarding sex-specific differences in surgically managed patients with IE still persists.

The main findings of our study are the following: (1) although some sex-differences emerged in terms of clinical presentation, risk profile, and anatomical distribution of the infection, males and females shared several clinical characteristics; (2) women tended to undergo surgery less frequently compared to men; (3) female sex was not associated with higher in-hospital mortality or higher incidence of the secondary endpoint.

Consistent with previous studies, IE was more frequently observed in male patients (20, 21). This is likely caused by a combination of biological characteristics, predisposing cardiac conditions, risk factors, and a more frequent use of invasive cardiac procedures (1). Several studies have identified that female patients affected by IE are generally older than male patients (6, 7, 9), with some authors hypothesizing that later onset may be attributed to the protective role of oestrogens against endothelial damage (12). In our study, we did not find any statistically significant difference in terms of median age of IE onset between groups. Furthermore, we found that mitral valve involvement was more frequent in women compared to men. Sex-related differences in native valve disease are well recognized, with women more commonly affected by pre-existing mitral valve abnormalities (22). Another noteworthy finding was that women in our cohort were more likely to have undergone TAVI prior to diagnosis of IE, reflecting the higher overall incidence of TAVI in female sex, as previously reported, irrespective of age (23). However, when focusing specifically on IE occurring after TAVI, previous studies have not identified significant sex-related epidemiological differences (24).

One of the most important findings of our study was the disparity in surgical access between sexes, with women undergoing surgery less frequently than men, which is consistent with previous reports (7, 9). However, in our cohort female sex did not emerge as an independent predictor of surgical access after adjusting for age, comorbidities, and surgical risk, suggesting that observed disparity may be driven by differences in clinical profiles and perceived operative risk rather than sex *per se*. On the other hand, well-established factors associated with increased surgical risk and poor prognosis in IE, such as higher Charlson Comorbidity Index (CCI), dementia, neurologic risk, and *Staphylococcus aureus* infection (1, 25), emerged as independent predictors of reduced surgical access in our study.

Some authors have suggested that disparities in surgical treatment may be related to a higher perceived operative risk in female patients, particularly given that validated risk stratification tools, such as the IE-STS score, include female sex as an independent risk variable. In our cohort, women exhibited significantly higher IE-STS Morbidity and Mortality scores, despite largely comparable baseline comorbidities between the sexes. However, other commonly used risk models, such as EuroSCORE II and APORTEI, showed no significant differences between groups. The relatively favourable outcomes observed in our cohort suggest that the IE-STS score may overestimate surgical risk in women underscoring the need for careful interpretation of validated risk scores that include sex as a variable, as they may not fully capture individual patient complexity and could inadvertently contribute to more conservative management strategies in women (26, 27).

Current literature presents conflicting evidence regarding the impact of sex on IE outcomes. While some studies suggest that females have higher mortality rates (9, 10) and are less likely to undergo surgical interventions (7, 8), others report no significant differences in surgical referral (28) or mortality (11–13). In our cohort, no significant differences between the two groups were observed in terms of early postoperative outcomes, including bleeding, intubation hours, acute kidney injury and cardiac tamponade. Third degree AV block emerged as the only statistically significant difference between the sexes, which could be explained by the more frequent mitral valve involvement among women. Approximately half of these blocks were permanent and required pacemaker implantation, with a higher incidence of device implantation also observed in the female cohort.

Patients with IE undergoing cardiac surgery have a reported in-hospital mortality of 7%–27% (22). Most studies on surgically treated patients with IE report increased short-term mortality in women. In a retrospective study carried out on 81.942 patients with diagnosis of IE, Bansal et al. documented a higher in-hospital mortality in female patients undergoing cardiac surgery (9.94% vs. 6.99% in male patients, *p* < 0.001) (9). Accordingly, in a retrospective observational study enrolling 413 patients, Friedrich et al. observed a higher 30-day mortality (26.7% in women vs. 14.9% in men, *p* = 0.007), with female sex being recognized as an independent early mortality predictor among surgically treated patients (10). Dohmen et al. reported similar mortality rates in patients undergoing aortic valve surgery for IE (23% vs. 15%, *p* = 0.01) (13). Conversely, our results showed similar rates of in-hospital mortality between women (9.1%) and men (13.7%), with no differences up to 1-year follow up. In multivariable analysis, female sex was not identified as an independent predictor of in-hospital mortality, suggesting that female patients potentially derive substantial benefit from surgical treatment. The absence of excess postoperative mortality or morbidity in women supports the hypothesis that surgery may attenuate, or even eliminate, the treatment gap traditionally attributed to female sex in IE. From a clinical perspective, this reinforces the importance of ensuring equitable and timely access to surgery for women with guideline-based indications, as disparities in treatment allocation, rather than intrinsic biological differences, may play a more relevant role in determining outcomes.

Longer diagnosis-to-surgery time emerged as an independent and protective predictor towards in-hospital mortality (OR: 0.877, *p* = 0.033). At first glance, this finding may appear counterintuitive, as recent evidence emphasizes the importance of early surgical intervention. However, this trend is heavily influenced by a treatment selection bias: patients undergoing immediate surgery are typically those presenting in critical, unstable conditions (e.g., cardiogenic shock or refractory heart failure), which inherently predisposes them to worse outcomes. Conversely, clinically stable patients can safely wait for optimized scheduling, paradoxically associating longer delays with survival. As this parameter is highly dependent on individual clinical status, it cannot be generalized as a uniform mortality predictor without extensive patient-level contextualization.

## 5 Conclusions

In conclusion, our findings may have important clinical implications for the management of IE in women. First of all they challenge the assumption that female sex is associated with worse surgical outcomes. Second, they highlight the need for careful individualized clinical judgment and multidisciplinary discussion within the Endocarditis Team to prevent potential treatment disparities. Third, they suggest that validated risk stratification tools that incorporate sex as a variable should be interpreted with caution and not used as the sole basis for surgical decision-making.

The observed disparity in surgical access, despite comparable outcomes, suggests that implicit biases may influence clinical decision-making. Future multicentre studies with larger sample sizes and longer follow-up periods are needed to confirm these findings and further elucidate the mechanisms underlying sex-related differences in IE outcomes.

## 6 Study limitations

Several limitations of this study should be acknowledged. First, the single-centre design may limit generalizability, although the prospective nature and multidisciplinary approach enhance internal validity. Second, the relatively small sample size may have limited statistical power to detect differences in subgroup analyses. Third, despite prospective data collection, some residual confounding may exist due to unmeasured variables such as socioeconomic factors, patient preferences, subtle differences in clinical presentation and lack of adjustment for time to antibiotic treatment before surgery. Fourth, the study period (2023–2025) represents a contemporary cohort, but temporal trends and evolving practice patterns may affect comparability with historical data. Fifth, the analysis of surgical decision-making, while comprehensive, cannot fully capture the complexity of individual clinical discussions and patient-specific factors that influence surgical candidacy including patients preferences, frailty assessments, and social support systems. Finally, the 1-year follow-up, while adequate for assessing medium-term outcomes, does not capture long-term survival or late complications.

## Data Availability

The raw data supporting the conclusions of this article will be made available by the authors, without undue reservation.

## Supplementary material

Supplementary Figure S1 Kaplan–Meier survival curves for 6-months mortality stratified by sex.

Supplementary Figure S2 Kaplan–Meier survival curves for 1-year mortality stratified by sex.

Supplementary Figure S3 Pearson correlation matrix of in-hospital mortality potential clinical predictors. The matrix illustrates the strength and direction of linear relationships between potential clinical predictors and in-hospital mortality. Coefficients (r) are shown within each cell, with color intensity representing the magnitude of the correlation, ranging from −1 (negative correlation; blue) to +1 (positive correlation; red).
