# Supplementary figures and images for: Female sex is not associated with worse surgical outcomes in infective endocarditis: a prospective study disproving a common assumption
Source: Front Cardiovasc Med. 2026 Jun 29;13:1883895. doi: 10.3389/fcvm.2026.1883895 (PMC13357413; doi:10.3389/fcvm.2026.1883895)

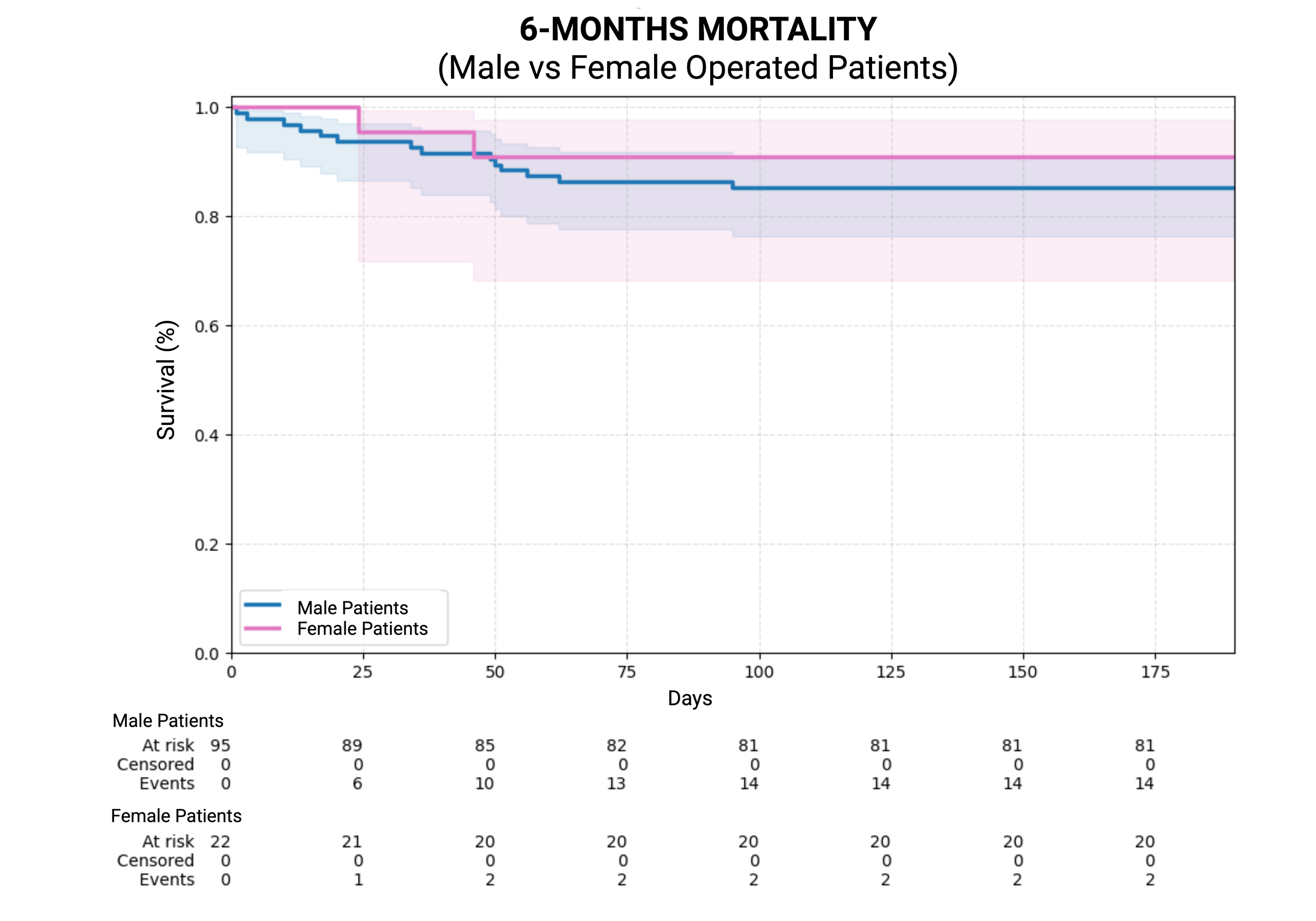

Supplement: Supplementary file 1 — Supplementary Figure S1 Kaplan–Meier survival curves for 6-months mortality stratified by sex. [file Image1.jpeg]

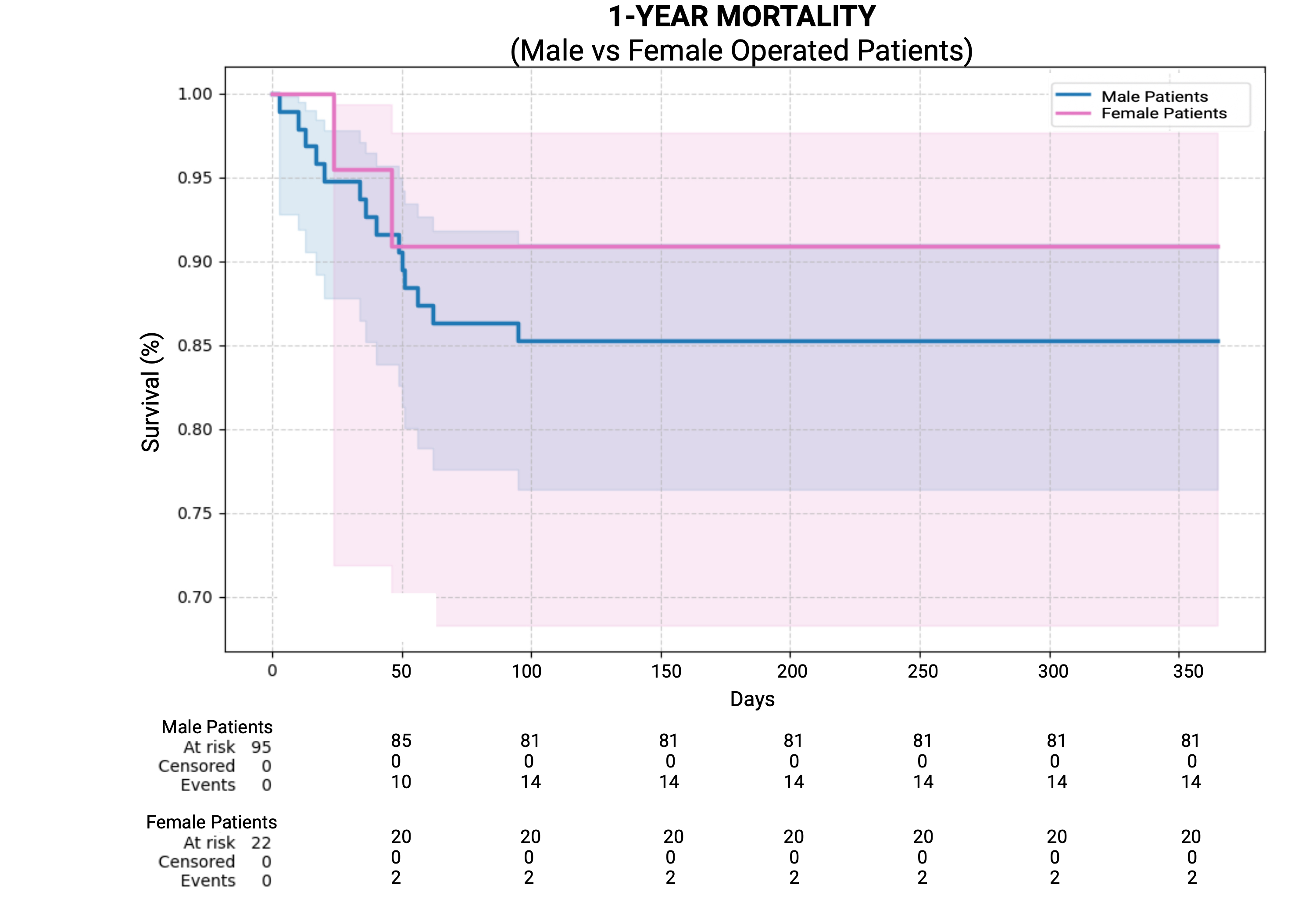

Supplement: Supplementary file 2 — Supplementary Figure S2 Kaplan–Meier survival curves for 1-year mortality stratified by sex. [file Image2.jpeg]

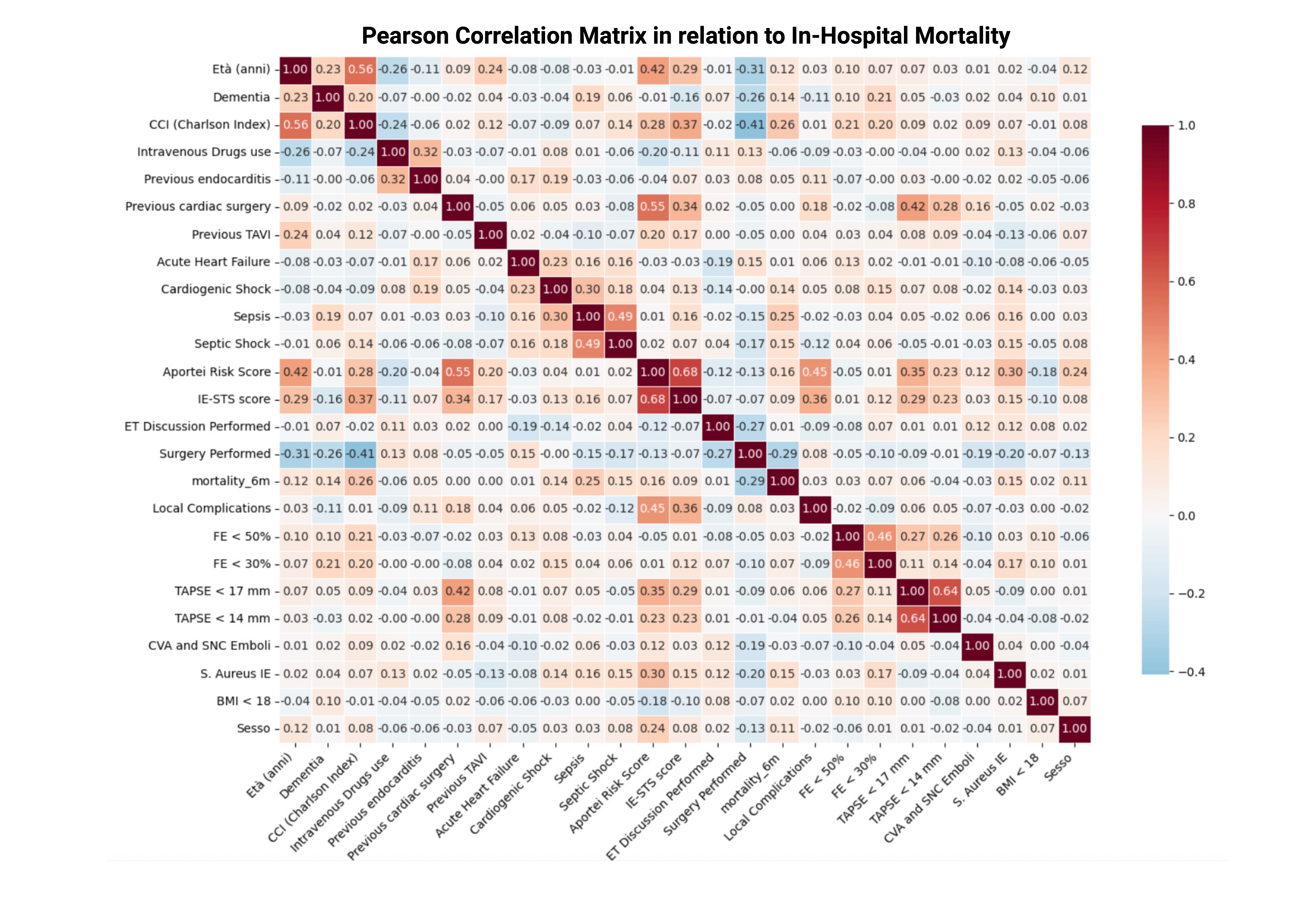

Supplement: Supplementary file 3 — Supplementary Figure S3 Pearson correlation matrix of in-hospital mortality potential clinical predictors. The matrix illustrates the strength and direction of linear relationships between potential clinical predictors and in-hospital mortality. Coefficients (r) are shown within each cell, with color intensity representing the magnitude of the correlation, ranging from −1 (negative correlation; blue) to +1 (positive correlation; red). [file Image3.png]
